# Hemostasis assessment in Fontan patients using the new thrombodynamics test

**DOI:** 10.1186/s43044-023-00365-2

**Published:** 2023-05-08

**Authors:** Anton V. Minaev, Dmitry K. Gushchin, Dmitry V. Kovalev, Bupe M. Mwela

**Affiliations:** 1 Congenital Heart Diseases Department, A.N. Bakoulev National Medical Research Center of Cardiovascular Surgery, Moscow, Russia; 2 Peoples’ Friendship University of Russia, Moscow, Russia

**Keywords:** ACHD, Fontan, Hemostasis, Thromboembolic events, Case report

## Abstract

**Background:**

Thromboembolic events are a well-known risk for Fontan patients and often lead to morbidity and mortality in cyanotic patients and patients with a single ventricle. Coagulopathy and thrombophilia, in addition to disturbed Fontan blood flow and endothelial injury, are major contributors to thromboembolic complications. However, there is currently no consensus regarding the optimal medication to prevent or treat these events. Identification of coagulation disorders is therefore crucial for selecting appropriate management strategies and evaluating long-term outcomes in these patients.

**Case presentation:**

We present the case of a 35-year-old male who underwent the Fontan procedure with a total cavopulmonary modification during childhood due to tricuspid atresia. He was admitted with complaints of headaches and visual disturbances, but no clear cardiovascular cause was identified. Standard coagulation test parameters were normal, but the thrombodynamics test indicated severe hypercoagulation and spontaneous clot formation. Anticoagulation therapy was initiated, and a subsequent thrombodynamics assay showed normalization of the coagulation parameters. The patient remained asymptomatic during the six-month follow-up period.

**Conclusions:**

The thrombodynamics test is a valuable tool for the diagnosis of coagulation disorders, as it can assess coagulation parameters and clot growth in vitro. This method can also aid in the optimization of antithrombotic therapy. The presented clinical case highlights the potential use of the thrombodynamics test in Fontan patients to diagnose coagulation disorders and improve long-term outcomes.

## Background

The Fontan procedure is the most common method of choice in surgery for patients with single-ventricular physiology. A number of children with a single ventricle have grown into adulthood with Fontan circulation. This group of children has increased over the past decades to 100,000 patients worldwide. Long-term survival with Fontan circulation has also increased and exceeded 80% by 20 years of age [1]. But there are many threatening factors in Fontan patients, one being thromboembolic events. The incidence of thromboembolic complications is different according to recent studies and rates, from 2 to 25% by 10 years, or between 0.74 and 5.2% per patient-year, which thereafter extends into adulthood [1, 2]. Thromboembolic complications are related not only to the distribution of Fontan blood flow, anatomical features and endothelial injury, but also to coagulopathy and coagulation abnormalities. Thus, one of the most relevant topics is complete diagnosis of coagulation disorders and control of antithrombotic medication.

## Case presentation

The thrombodynamics assay (Hemacore corporation, Russia) is a global method for the qualitative and quantitative assessment of plasma hemostasis. The main difference and principle of the test is to possibly stimulate the local vessel wall damage and thrombus formation in real time while monitoring the process. The preliminarily prepared plasma sample is placed into a special measuring cuvette in the thrombodynamics recording system. Then, an activator is introduced into the cuvette, which has a coating applied to one end containing lipids and tissue factor. This activates the coagulation process and the growth of fibrin clot begins from the end of the inserted activator. The chamber is illuminated with red (625 nm) radiation. Fibrin clot growth is detected by scattering red light, and the process is recorded using a digital camera for 30 min [3, 4]. Based on the obtained images, the device calculates quantitative indicators of the spatio-temporal dynamics of fibrin clot growth: from the time of initialization of measurement until the beginning of the clot growth (Tlag; N: 0.6–1.5 min), the initial velocity of clot growth (Vi; N: 38–56 um/min), the velocity of clot growth (V; N: 20–29 um/min), clot size (CS, um), clot density (D; N: 15,000–32,000 c.u.), and the duration of spontaneous clot formation (Tsp, min; N: abs.). Using this method, the thrombodynamics assay can visualize clot growth in vitro and provide information about the resulting coagulation state without the details of every separate coagulation stage.

**Fig. 1:**
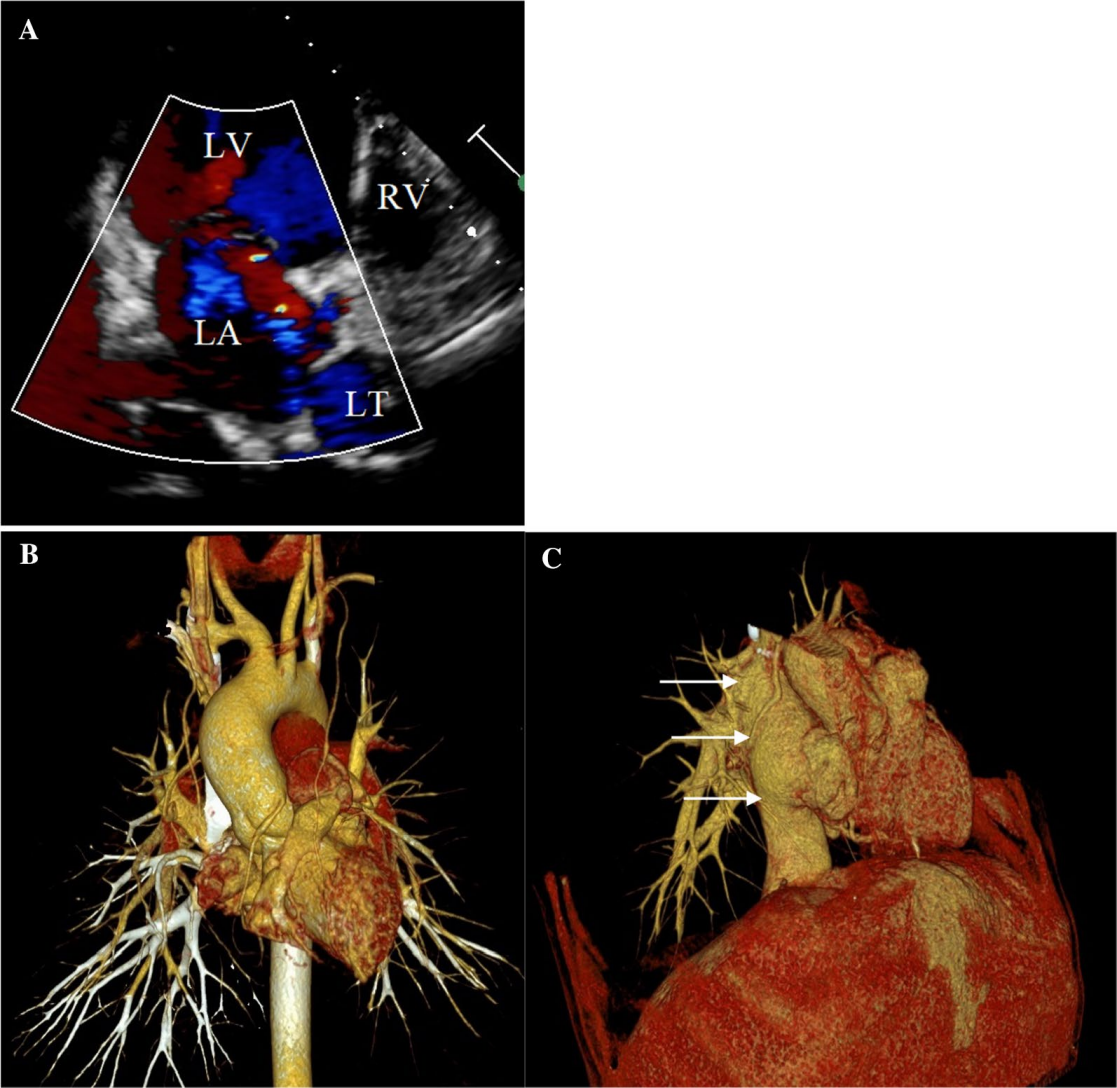
Echocardiogram and computed tomography. **A** 4 chamber view (LV—left ventricle, RV—right ventricle, LA—left atrium, LT—lateral tunnel), **B** anterior view, tricuspid atresia, **C** lateral view, total cavopulmonary anastomosis (lateral tunnel, white arrows)

**Fig. 2:**
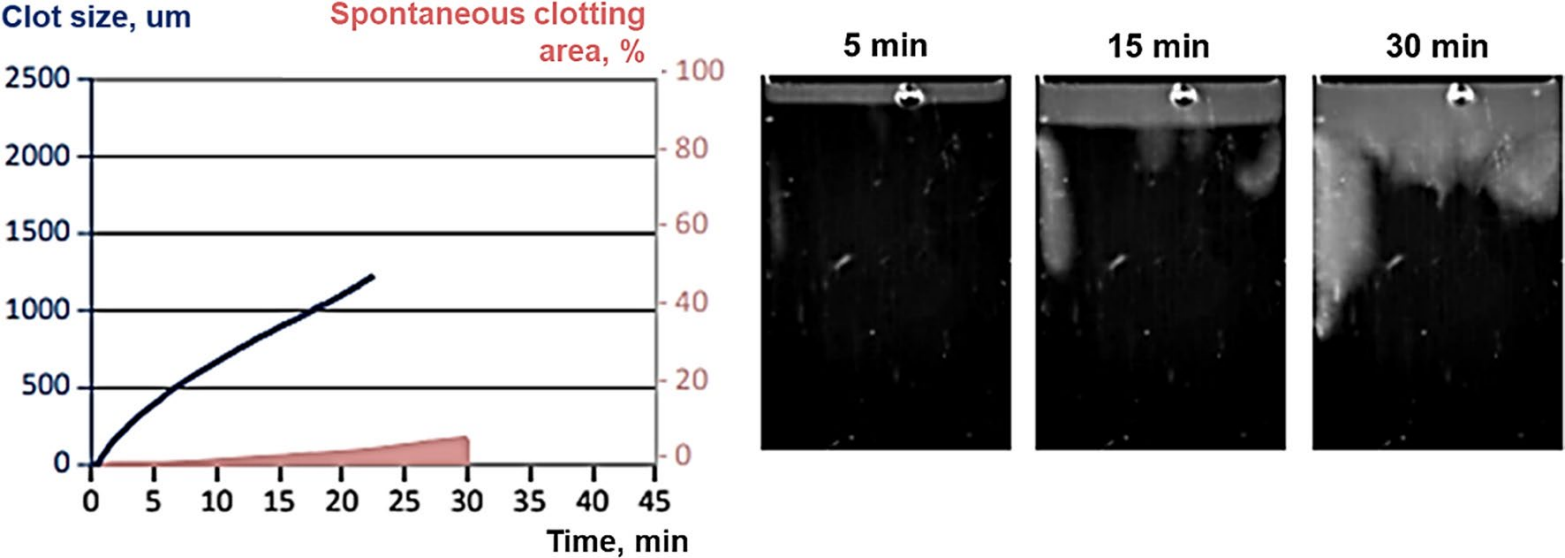
Thrombodynamics result before medication. The left diagram demonstrates high velocity of clot growth and formation of spontaneous clots. The right picture shows this process

We present a case report of a 35-year-old adult male, born with tricuspid atresia. In early childhood, he underwent a palliative systemic-to-pulmonary artery shunt and at 9 years old—the Fontan procedure with a total cavopulmonary modification (lateral tunnel). On admission, the patient had NYHA class II, episodes of headaches and visual disturbance which presented with an aura, and episodes of flashing lights. The symptoms were said to have been noticed for the last two years occurring once or a few times per month. No medication was prescribed. Echocardiography showed preserved ejection fraction, with no obstructions or valve insufficiency. Sinus rhythm was noted on ECG, but Holter-monitoring demonstrated sinoatrial node dysfunction with a decreased heart rate up to 20 beats per minute prevalence mainly at night. Cardiac catheterization showed normal mean pulmonary artery pressure (13 mmHg) and optimal pulmonary vascular resistance. Computed tomography showed absence of fenestration or shunts leading to embolism (Fig. 1). Further heart rhythm monitoring made it possible to establish that there was no connection between the symptoms and bradycardia. Laboratory results consisted of subnormal BNP level (37 pg/ml), normal biochemistry profile and normal standard coagulation test with INR 1.1, activated partial thromboplastin time 32 s; fibrinogen 2,8 g/l; antithrombin III 110%. Thrombodynamics method demonstrated that the velocity of clot growth (V) was 43,3 mcm/min (N: 20–29 mcm/min), the initial velocity of clot growth (Vi) was 66,4 mcm/min (N: 38–56 mcm/min), and spontaneous clot formation time (Tsp) was 25,3 min, the time from initialization of measurement until the beginning of clot growth (Tlag) and clot density (D) were within the normal range. It was impossible to measure clot size because of spontaneous clots. Presented data are suggestive of severe hypercoagulation hemostasis state with formation of spontaneous clots (Fig. 2).

**Fig. 3:**
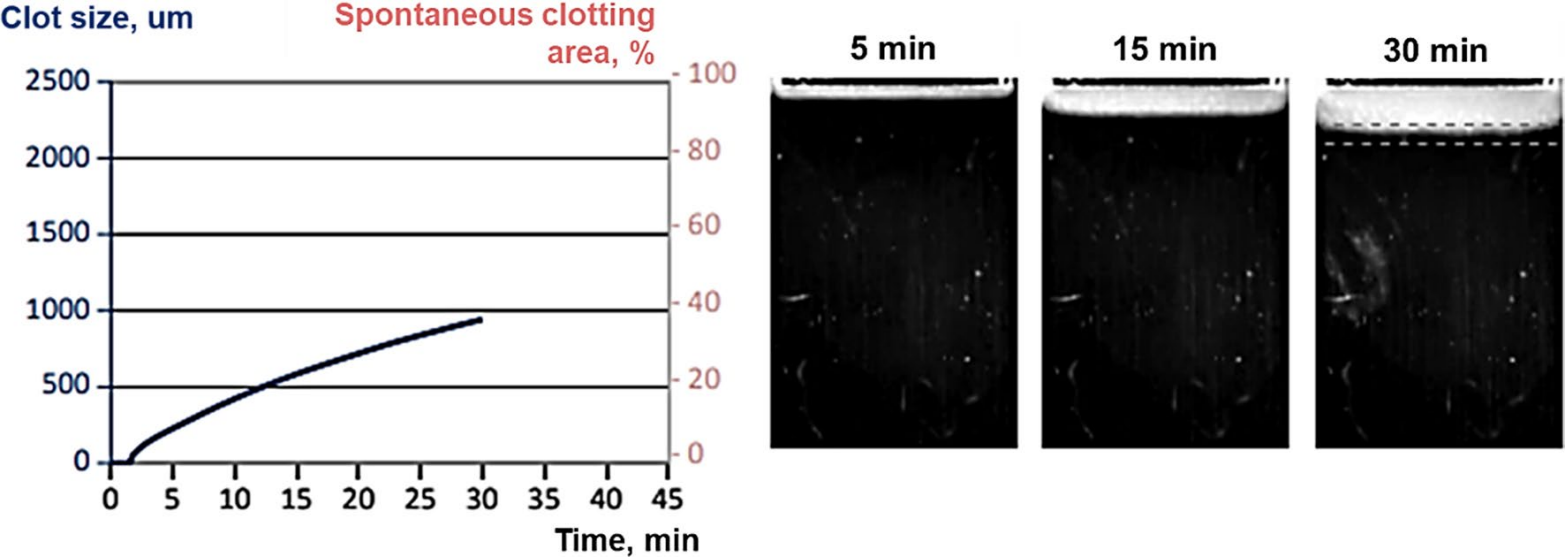
Control thrombodynamics with anticoagulation therapy. The left diagram demonstrates normal velocity of clot growth and absence of spontaneous clots. The right picture shows this process

The patient underwent epicardial pacemaker implantation and was treated with rivaroxaban 15 mg daily starting from day two post-operation. Control thrombodynamics assay showed normalization of the coagulation parameters: Vst decreased to 24.5 um/min (N: 20–29 mcm/min), Vi decreased to 41.1 um/min (N: 38–56), and no spontaneous clots (N: abs.) (Fig. 3). However, an increase of Tlag up to 2 min was noted and was as a result of rivaroxaban therapy. CS and D were within the normal range. For a period of six months, the patient had no symptoms or significant restrictions of physical activity (NYHA class I).

## Discussion

Today, Fontan surgery plays a leading role in the treatment of patients with univentricular circulation. This palliative operation aims to lead the systemic venous blood flow directly to the lungs, separating the functional single ventricle to support the systemic circulation and improve the patient’s quality of life. Long-term survival after Fontan procedure increases to 90% by 18 years old (upon reaching adulthood). At the same time, it leads to increased incidence of late Fontan-associated complications. One of the most significant is thromboembolic complications. The incidence of thrombosis and thromboembolic events among Fontan patients is high (from 2 to 25% at 10 years or between 0.74 and 5.2% per patient-year according to different studies) with the highest values being in the first 3–12 months after the operation, which extends into adulthood [1, 5–7]. Regardless of occurrence, thromboembolic events can vary in character, severity, symptoms, clinical manifestations, may be local or massive, and lead to pulmonary embolism or ischemic stroke [8, 9].

The mechanism of thromboembolism after Fontan surgery is characterized through Virchow’s triad: blood flow stasis, endothelial injury, disbalance of clotting factors and platelet activity [10]. All these factors can exist in Fontan circulation and determine necessity of thromboprophylaxis [11–14]. According to the last European Guidelines for the management of adult congenital heart disease, anticoagulation is indicated in the presence, or with a history, of atrial thrombus, atrial arrhythmias, or thromboembolic events. However, some complications can be silent or with non-specific symptoms which may be difficult to detect. Apart from that, thromboembolic events can develop without any history, especially in adults. That’s why there is no clear consensus about the choice of thromboprophylaxis, as well as its timing.

## Conclusions

The results of thrombodynamics assays help to understand current coagulation status in Fontan patients with or without thromboprophylaxis, the measurement of clot growth parameters, clot size and density. The presence of spontaneous clots is important for choosing a strategy for coagulation management. As was demonstrated, regular monitoring of hemostasis system parameters is necessary with comprehensive assessment, considering the low sensitivity of hemostasis clotting tests. The thrombodynamics method can become an important tool for the timely diagnosis of hypo- and hypercoagulable status with a personalized targeted approach to medication.

## Data Availability

The datasets supporting the conclusions of this article are included within the article.

## References

[1] Heidendael JF, Engele LJ, Bouma BJ, Dipchand AI, Thorne SA, McCrindle BW, Mulder BJM (2022). Coagulation and anticoagulation in fontan patients. Can J Cardiol.

[2] Deshaies C, Hamilton RM, Shohoudi A (2019). Thromboembolic risk after atriopulmonary, lateral tunnel, and extracardiac conduit fontan surgery. J Am Coll Cardiol.

[3] Dashkevich NM, Ovanesov MV, Balandina AN, Karamzin SS, Shestakov PI, Soshitova NP, Tokarev AA, Panteleev MA, Ataullakhanov FI (2012). Thrombin activity propagates in space during blood coagulation as an excitation wave. Biophys J.

[4] Lipets EN, Ataullakhanov FI (2015). Global assays of hemostasis in the diagnostics of hypercoagulation and evaluation of thrombosis risk. Thromb J.

[5] Balling G, Vogt M, Kaemmerer H, Eicken A, Meisner H, Hess J (2000). Intracardiac thrombus formation after the Fontan operation. J Thorac Cardiovasc Surg.

[6] McCrindle BW, Manlhiot C, Cochrane A, Roberts R, Hughes M, Szechtman B, Weintraub R, Andrew M, Monagle P (2013). Fontan Anticoagulation Study Group Factors associated with thrombotic complications after the Fontan procedure: a secondary analysis of a multicenter, randomized trial of primary thromboprophylaxis for 2 years after the Fontan procedure. J Am Coll Cardiol.

[7] Faircloth JM, Roe O, Alsaied T, Palumbo JS, Vinks A, Veldtman GR (2017). Intermediate term thrombotic risk in contemporary total cavopulmonaryconnection for single ventricle circulations. J Thromb Thrombolysis.

[8] Egbe AC, Connolly HM, Niaz T, Yogeswaran V, Taggart NW, Qureshi MY (2017). Prevalence and outcome of thrombotic and embolic complications in adults after Fontan operation. Am Heart J.

[9] Monagle P, Karl TR (2001). Thromboembolic problems after the Fontan operation. Pediatr Card Surg Annu Semin Thorac Cardiovasc Surg.

[10] Attard C, Huang J, Monagle P, Ignjatovic V (2018). Pathophysiology of thrombosis and anticoagulation post Fontan surgery. Thromb Res.

[11] Monagle P, Chan AKC, Goldenberg NA, Ichord RN, Journeycake JM, Nowak-Göttl U, Vesely SK (2012). Antithrombotic therapy in neonates and children: Antithrombotic Therapy and Prevention of Thrombosis, 9th ed: American College of Chest Physicians Evidence-Based Clinical Practice Guidelines. Chest.

[12] Potter BJ, Leong-Sit P, Fernandes SM (2013). Effect of Aspirin and warfarin therapy on thromboembolic events in patients with univentricular hearts and Fontan palliation. Int J Cardiol.

[13] Alsaied T, Alsidawi S, Allen CC, Faircloth J, Palumbo JS, Veldtman GR (2015). Strategies for thromboprophylaxis in Fontan circulation: a meta-analysis. Heart.

[14] Stout KK, Daniels CJ, Aboulhosn JA (2018). AHA/ACC guideline for the management of adults with congenital heart disease: a report of the American College of Cardiology/American heart association Task Force on clinical practice guidelines. J Am Coll Cardiol.

